# Does local steroid injection have a prognostic value for endoscopic plantar fascia release in chronic plantar fasciopathy?

**DOI:** 10.1186/s12891-025-08816-4

**Published:** 2025-06-09

**Authors:** Mohamed A. Mohamed, Mohammed S. Redwan, Hassan H. Noaman, Moustafa Elsayed

**Affiliations:** Department of Orthopaedics and Traumatology, Sohag Faculty of medicine, Sohag University, Sohag, Egypt

**Keywords:** Heel pain, Plantar fasciitis, Steroid injection, Endoscopy, Plantar fascia release

## Abstract

**Purpose:**

To determine if previous local steroid injection can alter the patient response to endoscopic plantar fascia release.

**Methods:**

It is a prospective non-randomized comparative study of 100 adult patients, suffered from plantar fasciopathy for at least one year, and had reported either temporary or no response to two or more of conservative treatments, including local corticosteroid injection. Enrolled patients were non-randomly allocated by convenience sampling into two groups. The first 50 patients who reported improvement in response to local corticosteroid injection were allocated to Group (A) The first 50 patients who did not report any improvement after injection were allocated to group (B) Both groups underwent endoscopic plantar fascia release. Clinical evaluation was carried out using the visual analogue scale (VAS), American Orthopaedic Foot and Ankle-Hindfoot Scale (AOFAS) and Patient self-assessment (Roles and Maudsley score) preoperatively and at 4, 8 weeks, 3, 6, 12, and 24 months postoperatively.

**Results:**

Both groups showed a statistically significant improvement in VAS for heel pain, AOFAS score, and Roles and Maudsley score. Group A demonstrated significantly better VAS, AOFAS, and self-assessment scores than group B at different follow-up intervals. Furthermore, trajectory analysis showed faster pain relief and functional recovery was observed in group A compared to group B.

**Conclusion:**

Patients who experienced temporary improvement after local corticosteroid injection had better clinical outcomes following endoscopic plantar fascia release.

**Clinical trial number:**

Not applicable.

## Introduction

Plantar fascia is substantial tissue band that connects the medial tubercle of the undersurface of the calcaneus to the metatarsophalangeal joints, forming the medial arch of the foot [1]. Plantar fasciopathy is actually a degenerative disorder that represents the root cause of 11–15% of all foot problems in both active and sedentary people [2]. Patients typically complain of heel pain when they bear weight, especially first thing in the morning and after a period of inactivity [3]. By passively hyperextending the metatarsophalangeal joints, one can mimic the pain by straining the plantar aponeurosis [4]. Other causes of heel pain like; fat pad atrophy, heel contusion, Tibialis posterior tendinitis, tarsal tunnel syndrome, and entrapment neuropathies of the first branch of the lateral plantar nerve should be distinguished from plantar fasciopathy [5]. Ultrasonography is a reference diagnostic tool. It is diagnostic when the plantar fascia insertion thickens by more than 5 mm [6].

Conservative treatments, including painkillers, local steroid injection, platelets-rich plasma injection, orthotics, plantar fascia stretching exercises, and shockwave therapy, are effective for the majority of people with plantar fasciopathy [4, 5]. Surgical treatment is indicated for patients who endure intractable pain and do not find relief through conservative care for at least 6 months [7]. Endoscopic plantar fasciotomy is a relatively new procedure developed by Barrett and Day [8]. This method has various significant benefits, including: minimum soft tissue dissection, excellent plantar fascia visualisation, precision in transecting only the medial third to one-half of the plantar fascia, minimum postoperative pain with an early return to full weight bearing, and a quicker return to activities and work are all desirable outcomes [9, 10].

Local steroid injection is one of the modalities frequently used in the treatment of plantar fasciopathy due to its beneficial long-term effects [11]. However some cases show relapse after variable time of improvement while others do not show any improvement after injection [5, 12]. Although there are some studies investigating the effect of previous local steroid injection on shoulder, hip and carpal tunnel surgeries [13–15], the relationship between patients’ response to previous local steroid injection and improvement after endoscopic plantar fascia release has not yet been investigated. These studies [13–15] found that local steroid injection has both diagnostic and prognostic effect in patients who underwent surgery.

The purpose of the present study was to investigate if patient’s response to previous local steroid injection is predictive of their response to endoscopic plantar fascia release as regards pain relief, functional recovery, and patient satisfaction. We hypothesized that previous temporary improvement in response to local corticosteroid injection is associated with favourable outcomes following endoscopic release.

## Materials and methods

This prospective comparative study was conducted between March 2022 and April 2024. A total of 100 patients; 18 years age or older, suffered from heel pain for at least one year and were diagnosed as plantar fasciopathy. In order to qualify, patients had to report either temporary or no improvement in response to local corticosteroid injection plus any of the following conservative measures: NSAIDs (nonsteroidal anti-inflammatory drugs), physical therapy, a workout routine, including stretches for the plantar fascia and the Achilles tendon, and orthotics. An informed consent was obtained from each participant and the study was approved by our Ethical Committee.

The diagnosis relied heavily on patient history and physical examination, Patients presented by progressive pain at the inferior and medial heel. Pain is characterized by being worse with the first few steps out of bed in the morning then decreases with walking. Tenderness over the plantar medial calcaneal tubercle at the site of the plantar fascial insertion was detected during examination. Passive dorsiflexion of the foot and toes was used as a provocative test as it can reproduce pain. All patients, however, had a calcaneus x-ray taken before surgery to prove if they had a heel spur or not. Age, gender, employment, affected side, length of symptoms, and prior steroid injections were noted for each patient. All patients had a history of local corticosteroid injections 3 months or more before operation in the form of 1 ml Betamethasone dipropionate (5 mg/ml) and Betamethasone sodium phosphate (2 mg/ml) combination with 0.5 ml 1% Lidocaine.

Criteria for exclusion were patients under the age of 18, patients with a history of tarsal tunnel syndrome, seronegative arthropathy, widespread polyarthritis, diabetes, limb length discrepancy, in-toeing, and neuromuscular issues, patients who have cancers, vascular anomalies, or neurological disorders on either side of the body, gastrocnemius or Achilles tendon contracture, fractures, deformities (including flat foot and high arched foot), or recent injuries to the foot, ankle, or both, and disorders of bleeding or active anticoagulant therapy.

Enrolled patients were non-randomly allocated by convenience sampling into two groups. The first 50 eligible patients who reported temporary clinical improvement, disappearance of clinical symptoms, in response to local corticosteroid injection, followed by relapse of symptoms, were allocated to Group (A) The first 50 eligible patients who did not report any clinical improvement after local corticosteroid injection were allocated to group (B) Both groups were treated with endoscopic plantar fascia release.

Preoperative and postoperative pain assessment, defined by morning agony, was carried using the 10-point visual analogue scale (VAS) where lower scores indicate less pain. Functional assessment was carried out using the 100-point American Orthopaedic Foot and Ankle-Hindfoot Scale (AOFAS) [16] where higher scores indicate better function. Patient self-assessment was carried out using the 4-point Roles and Maudsley score where a score of 1 indicates excellent response with no pain and full activity, and a score of 4 indicates poor response with pain-limiting activity [17]. Patients received evaluations at 4, 8 weeks, 3,6,12 and 24 months postoperatively.

### Operative technique

**Fig. 1:**
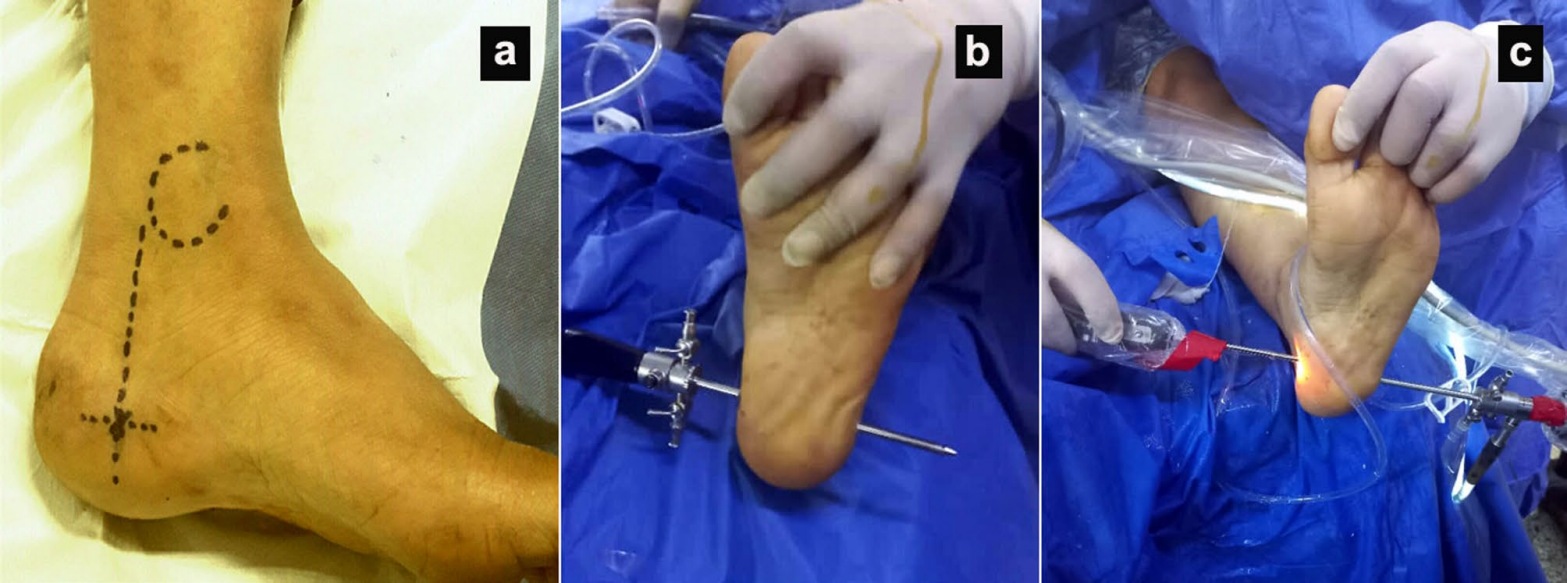
Intraoperative photos showing: (**a**) The medial portal was made by cutting a vertical line across the medial malleolus’ posterior border while holding the foot in a neutral position. (**b**) 5 mm cannula and blunt trocar are apparent transversing the heel and exiting from the lateral portal. (**c**) Insertion of the scope from lateral portal and the shaver from medial portal

**Fig. 2:**
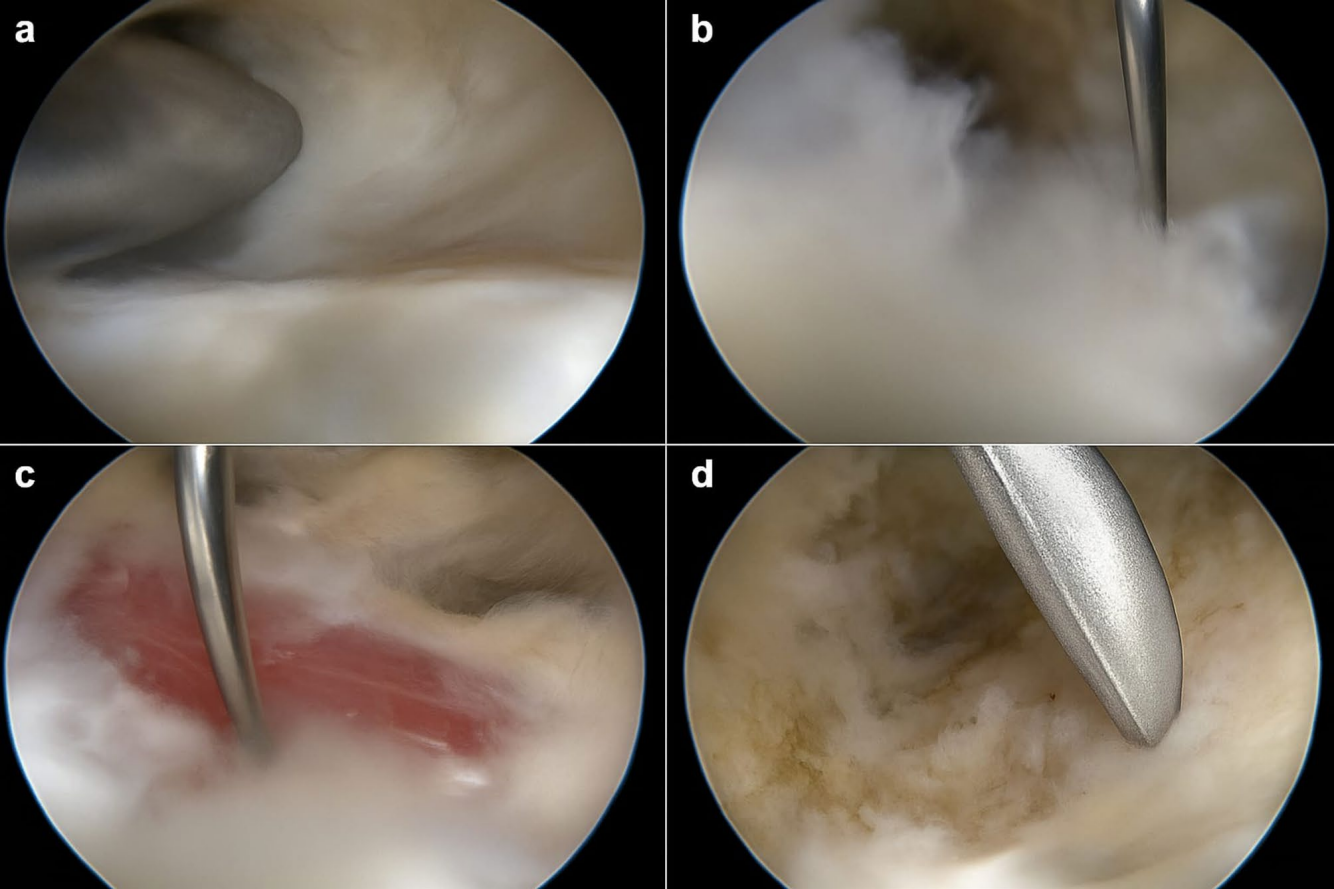
Endoscopic images showing: (**a**) Highlighting the plantar fascia’s gleaming fibers. (**b**) Insertion of a needle crossing the tunnel that serves as an identifier for the center of the plantar fascia. (**c**) Posterior leaflet debrided and the fibers of the abductor hallucis exposed. (**d**) The entire posterior leaflet was debrided up to bone

Prophylactic antibiotic was given an hour before operation. Under spinal anaesthesia, in supine position with the foot hanging off the surgical table, and a pneumatic tourniquet was applied to the thigh. A medial portal was made by cutting a vertical line across the medial malleolus’ posterior border while holding the foot in a neutral position (Fig. 1a). A 5 mm cannula trocar with a blunt tip was used to make a transverse tunnel in subcutaneous tissue just under plantar fascia. To access the cannula, a lateral portal was created (Fig. 1b). Then, a gauze tape was repeatedly passed between the medial and lateral portals to form a subcutaneous tunnel. The plantar fascia served as the tunnel’s ceiling. After creating a lateral incision, the cannula was inserted. The sheath was then put over the trocar through the lateral portal after the blunt trocar had been repositioned from medial to lateral portal. Then, a line was installed to bring in irrigation fluid at a pressure of 50–60 mmHg. The cannula was used to insert an endoscope (Fig. 1c). The subcutaneous tissue was debrided using a motorized shaver blade until the plantar fascia lustrous fibres were clearly seen (Fig. 2a). As shown in (Fig. 2b), the plantar fascia centre was marked by vertically puncturing the heel skin with a needle. Full thickness release up to half of plantar fascia, release finishes when abductor hallucis muscle fibres are visible (Fig. 2c). After exposing the bony plantar fascia attachment, with a motorised incisor blade, the entire posterior leaflet was debrided (Fig. 2d). Finally, we irrigate the tube, use 3 − 0 proline suture to close portals, dressing and a crepe bandage were applied.

### Post-operative follow-up

After surgery, patients received rest, limb elevation, and ice packs. Patients were given oral antibiotics, analgesics, and anti-oedematous medications and were released the same day or the first post-operative day. After two weeks of non-weight bearing, toe touch bearing is followed by full weight bearing, if tolerated. Sterile dressing was utilized every three days. Stitches were removed at outpatient clinic 10–14 days following operation. The first check-up was scheduled for the removal of stitches and for the start of weight bearing after two weeks. After 4 weeks, 8 weeks, 3 months, 6 months, one year and 2 years following surgery, the patients were checked for pain and functional improvement, based on the following: Morning pain ratings using VAS, AOFAS, and subjective patient assessment utilising Roles and Maudsley’s criteria.

### Statistical analysis

Statistical analysis was done by SPSS version 26.0 (IBM Inc., Chicago, IL, USA). Shapiro-Wilks test and histograms were used to evaluate the normality of the distribution of data. Qualitative variables were presented as frequencies and percentage and were analysed utilizing the Chi-square test. Quantitative parametric variables were presented as mean and standard deviation (SD) and compared between the groups A and B utilizing independent sample t test. Quantitative non-parametric data were presented as median and interquartile range (IQR) and were compared by Mann-Whitney U test. Friedman test was used to compare preoperative and postoperative measurements in each group at different follow-up intervals. Trajectory analysis was also carried out to demonstrate changes in outcome measures over time. Because three outcome measures were tested, a Bonferroni-adjusted *P* value of 0.0167 was calculated to declare statistical significance.

## Results

A total of 50 patients in each group were included in the study. Patients were followed for 2 years postoperatively. Three (6%) patients in group A and four (8%) patients in group B were lost to follow-up. Table 1 summarizes the baseline characteristics of enrolled patients. No statistically significant difference was observed between group as regards age, gender, occupation, body mass index, duration of symptoms, number of steroid injections, the presence of calcaneal spurs, and the side of heel pain.

**Table 1:** Baseline data of the studied patients (*n* = 100)

	Group A (*n* = 50)	Group B (*n* = 50)	*P* value
Age, years			0.332^*^
Mean ± SD	45.88 ± 8.71	47.5 ± 7.88	
Range	28–56	38–60	
Gender			0.072^**^
Male	20 (40%)	29 (58%)	
Female	30 (60%)	21 (42%)	
Occupation			0.102^**^
Housewife	34 (68%)	26 (52%)	
Worker	16 (32%)	24 (48%)	
BMI, kg/m^2^			0.736^*^
Mean ± SD	25.64 ± 3.01	25.8 ± 1.45	
Range	22–34	24–28	
Duration of Symptoms, years			0.065^*^
Mean ± SD	2.05 ± 0.38	1.93 ± 0.25	
Range	1.5–3	0.5–2	
Number of steroid injections			0.109^*^
Mean ± SD	1.38 ± 0.49	1.14 ± 0.93	
Range	1–2	1–3	
Calcaneal Spurs	40 (80%)	39 (78%)	0.806^**^
Involved Side			0.107^**^
Right	18 (36%)	26 (52%)	
Left	32 (64%)	24 (48%)	

SD, standard deviation; BMI, body mass index

^*^ Independent sample t test. ^**^ Chi-square test

### VAS for heel pain

**Fig. 3:**
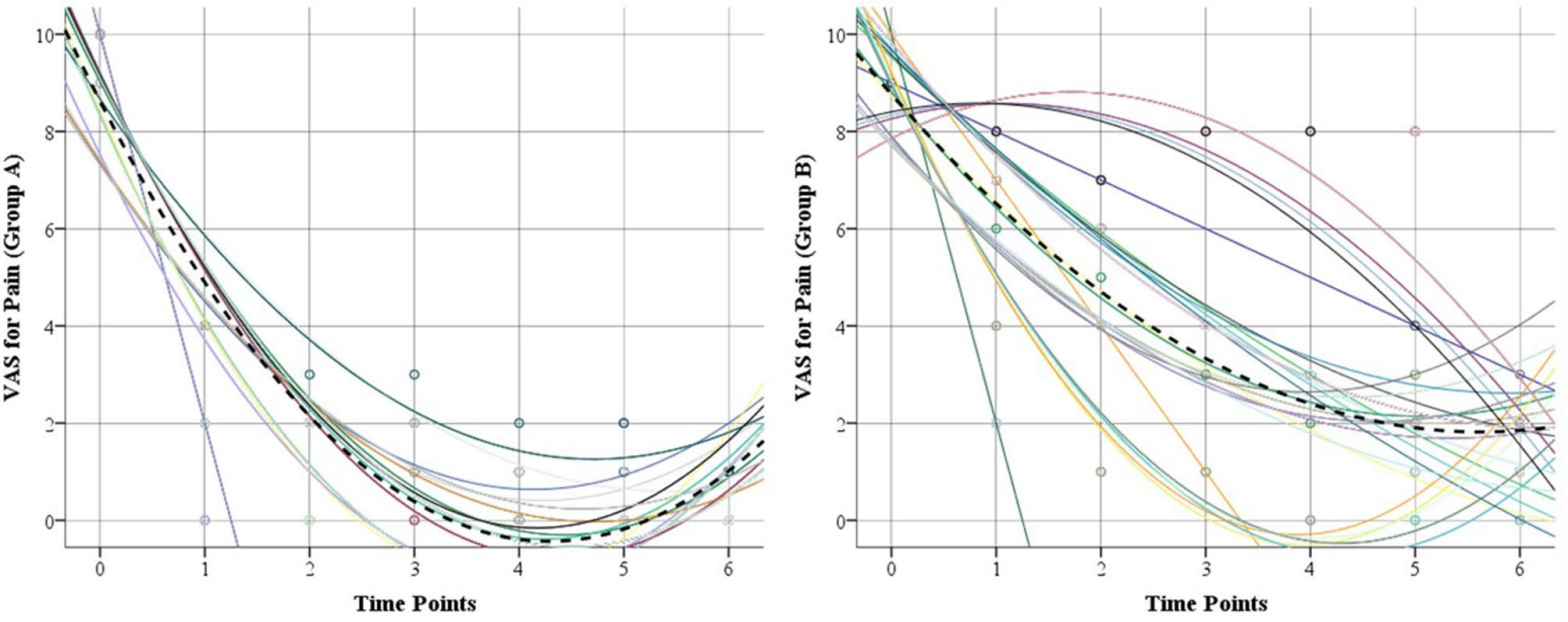
Trajectory analysis of changes in VAS in both groups during follow up

As shown in Table 2, the median baseline VAS for heel pain declined from 10 (IQR, 10–10) to 4 (IQR, 2–4) and from 10 (IQR, 9–10) to 6 (IQR, 4–7) at 4 weeks in groups A and B, respectively (*P* <.001). Pain relief was maintained in both groups throughout the follow-up period. At final follow-up, the median VAS declined to 0 (IQR, 0–1) in group A and 1 (IQR, 0–2) in group B. However, by running a trajectory analysis, different patterns of pain improvement have been observed where group B demonstrated rather slower pain reduction over time (Fig. 3). Pain levels were significantly lower in group A than group B at 4, 8 weeks, 3, 6, 12, and 24 monthspostoperatively (*P* <.001).

**Table 2:** Pain assessment by VAS for studied patients

	Baseline	4 weeks	8 weeks	3 months	6 months	12 months	24 months
Group A							
No. Patients	50	50	47	47	47	47	47
Median (IQR)	10 (10–10)	4 (2–4)	2 (2–2)	1 (0–1)	0 (0–0)	0 (0–1)	0 (0–1)
Group B							
No. Patients	50	50	46	46	46	46	46
Median (IQR)	10 (9–10)	6 (4–7)	4.5 (1–6)	4 (1–4)	3 (0–3)	2 (1–3.3)	1 (0–2)
*P* value^*^	0.055	< 0.001	< 0.001	< 0.001	< 0.001	< 0.001	< 0.001

IQR: interquartile range

^*^ Mann-Whitney U test

### AOFAS score

**Fig. 4:**
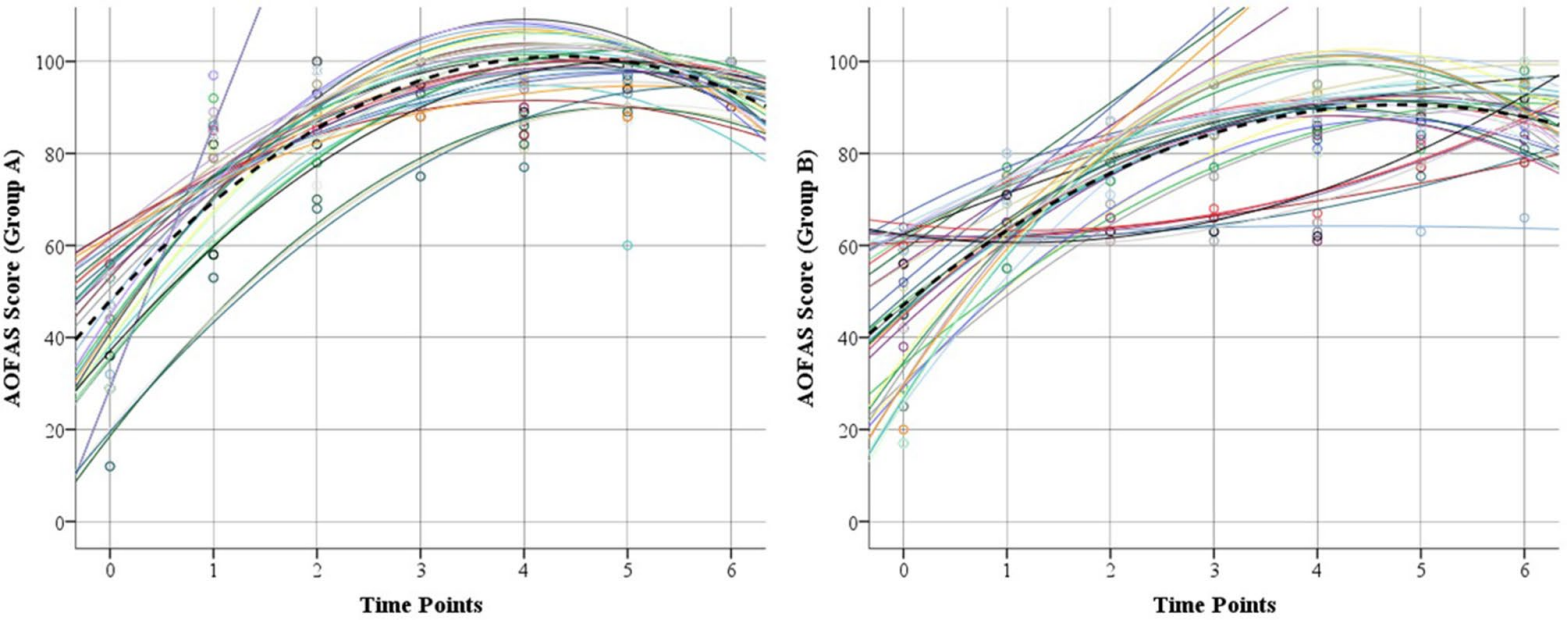
Trajectory analysis of changes in AOFAS in both groups during follow up

The median baseline AOFAS scores were 44 (IQR, 29–53) in group A and 43.5 (IQR, 25–56) in group B (*P* =.575). A statistically significant improvement in ankle and hindfoot function was observed at 4 weeks to a median score of 84 (IQR, 79–86) and 71 (IQR, 65–74.3) in groups A and B, respectively (*P* <.001). Functional improvement continued in both groups to a median of 100 (IQR, 98–100) in group A and 94 (IQR, 87.8–96) in group B at 2 years postoperatively. However, trajectory analysis showed a faster functional recovery in group A compared to group B (Fig. 4). Furthermore, group A demonstrated higher functional scores than group B at 4, 8 weeks, 3, 6, 12, and 24 months postoperatively (*P* <.001). Details of AOFAS score components are summarized in Table 3.

**Table 3:** AOFAS scores of the studied patients

	Baseline	4 weeks	8 weeks	3 months	6 months	12 months	24 months
**Group A**							
N	50	50	47	47	47	47	47
Total	44 (29–53)	84 (79–86)	90 (86–98)	98 (93–100)	90 (88– 100)	100 (96– 100)	100 (98–100)
•Pain	20 (0–20)	30 (30–30)	30 (30–40)	40 (40–40)	30 (30–40)	40 (40–40)	40 (40–40)
•Function	24 (22–31)	44 (44–47)	48 (47–50)	50 (48–50)	48 (47–50)	50 (48–50)	50 (50–50)
•Alignment	5 (5–5)	10 (5–10)	10 (5–10)	10 (10–10)	10 (10–10)	10 (10–10)	10 (10–10)
**Group B**							
N	50	50	46	46	46	46	46
Total	43.5 (25–56)	71 (65–74.3)	80 (74–82)	87 (77–95)	86.5 (80–93)	88.5 (84.8–93.3)	94 (87.8–96)
•Pain	0 (0–20)	30 (20–30)	30 (30–30)	30 (30–40)	30 (30–40)	30 (30–40)	40 (30–40)
•Function	31 (20–34)	39 (36–40)	40 (38–47)	47 (40–50)	46 (41–49)	47 (44–50)	48 (45–50)
•Alignment	5 (4–5)	5 (5–5)	10 (5–10)	10 (5–10)	10 (5–10)	10 (5–10)	10 (5–10)
*P* value^*^	0.575	< 0.001	< 0.001	< 0.001	< 0.001	< 0.001	< 0.001

Data are presented as median (interquartile range)

* Total AOFAS scores between Group A and B were compared using Mann-Whitney U test

**Table 4:** Patient Self-Assessment by roles and Maudsley score for studied patients

	Baseline	4 weeks	8 weeks	3 months	6 months	12 months	24 months
Group A							
No. Patients	50	50	47	47	47	47	47
Excellent	0 (0)	4 (8)	26 (55)	29 (62)	36 (77)	32 (68)	42 (89)
Good	0 (0)	36 (72)	21 (45)	18 (38)	11 (23)	15 (32)	5 (11)
Acceptable	0 (0)	10 (20)	0 (0)	0 (0)	0 (0)	0 (0)	0 (0)
Poor	50 (100)	0 (0)	0 (0)	0 (0)	0 (0)	0 (0)	0 (0)
Group B							
No. Patients	50	50	46	46	46	46	46
Excellent	0 (0)	0 (0)	0 (0)	13 (28)	15 (33)	20 (43)	20 (43)
Good	0 (0)	34 (68)	46 (100)	23 (50)	21 (46)	21 (46)	24 (52)
Acceptable	0 (0)	16 (32)	0 (0)	10 (22)	10 (22)	5 (11)	2 (4)
Poor	50 (100)	0 (0)	0 (0)	0 (0)	0 (0)	0 (0)	0 (0)
*P* value^*^	1.000	0.060	< 0.001	< 0.001	< 0.001	< 0.001	< 0.001

Data are presented as No. (%)

^*^ Mann-Whitney U test

### Roles and Maudsley score

**Fig. 5:**
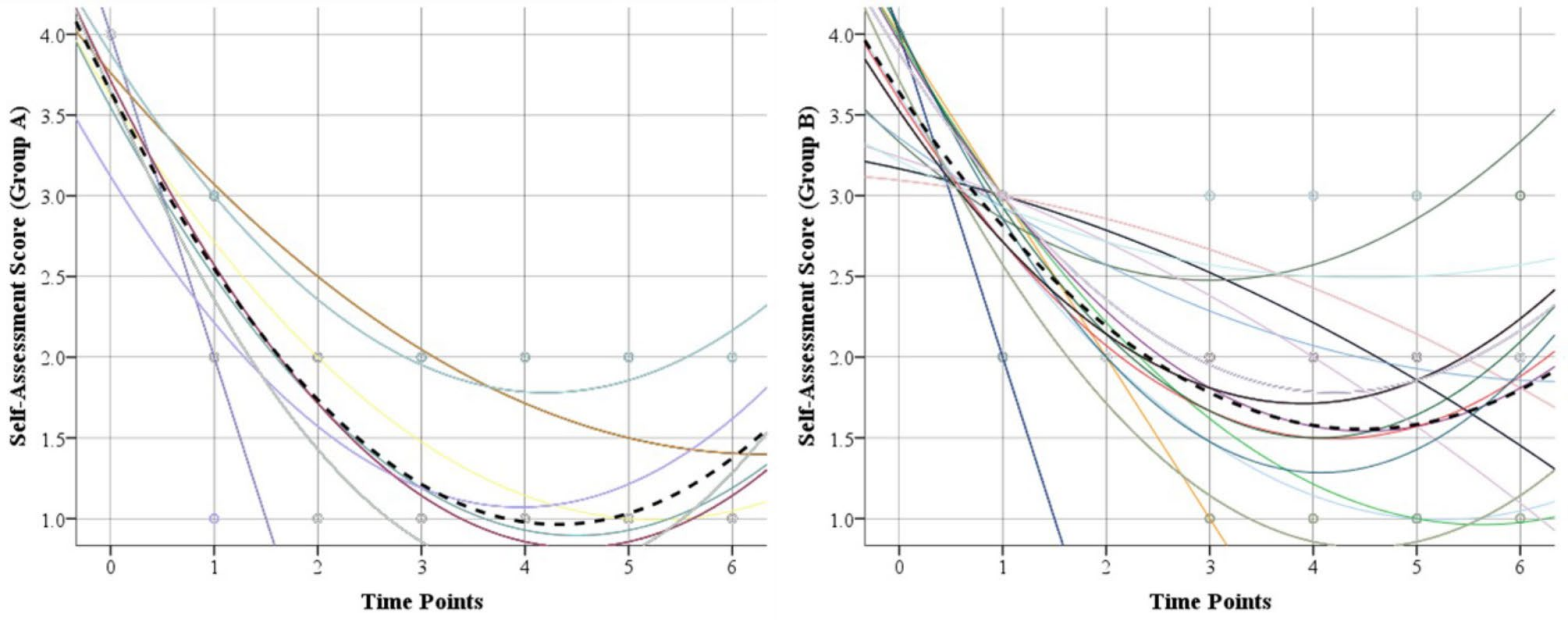
Trajectory analysis of changes in self-assessment in both groups during follow up

Preoperatively, all patients reported a median Roles and Maudsley score of 4 (poor) indicating pain-limiting activities. As shown in Table 4, a statistically significant improvement was observed at 4 weeks in patient self-assessment to a median score of 2 with 80% patients in group A reporting a good to excellent response and 68% in group B reporting a good response (*P* <.001). Both groups demonstrated further improvement in Roles and Maudsley score till 2 years postoperatively when 89% in group A reported an excellent response with a median score of 1, and 96% in group B reporting a good to excellent response with a median score of 2. Similar to VAS and AOFAS score, trajectory analysis showed a faster response to treatment as regards patient satisfaction in group A compared to group B (Fig. 5). Additionally, group A demonstrated better Roles and Maudsley scores than group B at 8 weeks, 3, 6, 12, and 24 months postoperatively (*P* <.001). No complications were recorded during or after the procedure in either group.

## Discussion

Several studies evaluated endoscopic plantar fascia release [8, 18, 19]. However, none of these studies, to our knowledge, have discussed patient response to previous local steroid injection. We noted that Local steroid injection has a prognostic value preoperatively in cases that will undergo endoscopic plantar fascia release; patients who temporary improved after steroid injection (Group A) showed better outcomes after endoscopic release as regard pain (VAS) (*P* <.001), function (AOFAS) (*P* <.001) and patient’s satisfaction (Roles and Maudsley) (*P* <.001) almost in all time intervals.

Our results are comparable to previous studies investigating shoulder, hip and carpal tunnel surgeries [13–15]. Mujahed T et al. [14] investigated the response to intra-articular steroid injection before hip arthroscopy and concluded that; intra-articular injection did aid in predicting 2-year outcomes of hip arthroscopy for femoroacetabular impingement and the result of a preoperative intra-articular injection can be a helpful clinical tool for surgical decision-making and counseling patients on expected outcomes after hip arthroscopy. Another study by Lim JT et al. [13] found that local steroid/ anesthetic injection is a useful tool both diagnostically and prognostically in patients with subacromial impingement syndrome who underwent arthroscopic subacromial decompression. In patients with a confirmed diagnosis but a negative response to injection there is still a significant improvement postoperative, but to a lesser degree than in those with a positive result to local injection.

Improvement of VAS score showed statically significant differences between both groups with significant improvement at 3 and 6 months for group A. Group A had statically significant improvement of AOFAS scores in comparison to Group B with a faster functional recovery in Group A. Adequate pain improvement after injection had a better and faster favourable functional outcome after endoscopy than no pain improvement. Faster improvement of AOFAS score in group A may be due to anti-inflammatory effect of steroid which may provide additional benefit for plantar fasciitis. The improvement in pain score was consistent with past literatures comparing preoperative steroid injection in several studies [14, 20]. Prior authors have noted improvement in AOFAS score after endoscopic plantar fascia debridement [21, 22]. In our present investigation, a statistically significant difference was observed after 4 weeks between 2 groups with higher satisfaction, as measured by Roles and Maudsley score, in group A. Previous studies supported the theory of improved satisfaction after endoscopic planter fascia release [22–24].

In their prospective study to evaluate the effectiveness and safety of endoscopic release of plantar fascia, El-Nagar et al. [19], after a six-month follow-up- found that the mean AOFAS preoperative score had increased from 51.36 to 89.44. The VAS score decreased from 85 before to surgery to 12.6. According to Roles and Maudsley criteria, 84 out of the patients received satisfactory results. Cottom JM et al. [25] investigated endoscopic plantar fascia debridement in 125 patients and found significant improvement in AOFAS (*p* =.035). On 70 patients with unilateral persistent plantar fasciopathy, a prospective comparative research between shock wave and endoscopic plantar fascia release was done by Radwan et al. [2] 31 patients made up the group for endoscopic release. The AOFAS score for this group was 44 before surgery, but it increased to 77 after one year. Patients who received good or excellent grades in the Roles and Maudsley criteria were 24/31 (77.6%) one year after surgery, which is comparable to our outcomes.

In order to govern the hindfoot during gait, the plantar fascia is crucial [26]. Although plantar fasciotomy reduces the height of the medial longitudinal arch and relieves focal strains on the plantar fascia’s origin, it also results in a less rigid and more malleable arch [27]. About 97% of 652 patients who underwent endoscopic plantar fasciotomy experienced alleviation from heel pain in a multi-surgeon prospective analysis [8]. But there was a 10% documented complication rate for lateral column overload with calcaneocuboid and mid-tarsal joint pain. It was discovered that when more than 50% of the plantar fascia was released, lateral column symptoms were more likely to develop independent of the surgical technique (endoscopic or open release) [28]. This was consistent with the findings of the current investigation, where just a 50% release was performed and no lateral column problems were noted.

Among our patients, 79 (79%) had calcaneal spurs. In a random sample of 1000 radiographs, one study found a 13% incidence of heel spurs, of which nearly a third were symptomatic [29]. Although removal of spur was not a common surgical procedure for treating plantar fasciopathy [30, 31], recent studies found that endoscopic calcaneal spur resection without plantar fascia release resulted in good outcomes and was sufficient to relieve symptoms and improve function [32, 33]. However, none of our patients underwent calcaneal spur resection.

Decompression of the Baxter nerve, which supplies the abductor digiti minimi nerve, was not a part of the surgery used in the current investigation. According to Cole et al. [34], there is an average distance of 10.5 and 12.3 mm between the release site and the lateral plantar nerve and the nerve to the abductor digiti minimi, respectively. Goff and Crawford [5] also showed that the plantar fascia could be safely divided using trustworthy markers. The posterior malleolar boundary and one centimetre from the plantar skin served as the reference lines in the current study.

Limitations of the current study included; small number of patients and short follow up period in comparison with other studies which had longer follow-up. Besides, there were no restrictions for patient enrolment as regard the technique of steroid injection, i.e., palpation-guided vs. ultrasound-guided. Also, we were not able to measure the patients’ response to previous local corticosteroid injection before enrolment. We choose the widely used AOFAS to evaluate the results, however, we had some difficulties to translate the score to our patients.

## Conclusions

From the current study we conclude that; patients who improved after local steroid injection had better clinical outcomes following endoscopic plantar fascia release which could be considered of prognostic value.

## Data Availability

The data sets used and/or analyzed during the current study are available from the corresponding author on request.
